# Modelling human immunodeficiency virus ribonucleic acid levels with finite mixtures for censored longitudinal data

**DOI:** 10.1111/j.1467-9876.2011.01007.x

**Published:** 2012-03

**Authors:** Bettina Grün, Kurt Hornik

**Affiliations:** 1 Johannes Kepler Universität LinzAustria; 2 Wirtschaftsuniversität WienAustria

**Keywords:** Censored response, EM algorithm, Finite mixture, Mixed effects model, Unobserved heterogeneity

## Abstract

The measurement of human immunodeficiency virus ribonucleic acid levels over time leads to censored longitudinal data. Suitable models for dynamic modelling of these levels need to take this data characteristic into account. If groups of patients with different developments of the levels over time are suspected the model class of finite mixtures of mixed effects models with censored data is required. We describe the model specification and derive the estimation with a suitable expectation–maximization algorithm. We propose a convenient implementation using closed form formulae for the expected mean and variance of the truncated multivariate distribution. Only efficient evaluation of the cumulative multivariate normal distribution function is required. Model selection as well as methods for inference are discussed. The application is demonstrated on the clinical trial ACTG 315 data.

## 1. Introduction

Dynamic models for human immunodeficiency virus ribonucleic acid (HIV RNA) levels are used to estimate viral dynamic parameters for the whole population and for each individual patient in an acquired immune deficiency syndrome clinical trial. These models provide a good understanding of the pathogenesis of HIV infection and the evaluation of anti-retroviral therapies. The analysis is based on the HIV type 1 (HIV-1) viral load which is primarily used to measure HIV-1 infection. However, accurate quantification is in general not possible for low levels, i.e. a lower detection limit is given under which no accurate measurement is possible and censored observations are reported.

Previous work on dynamic models for HIV RNA levels used a variant or simplification of non-linear mixed effects models while accounting for censored data and applied the method proposed to a (subset) of the HIV-1 viral load data from clinical trial ACTG 315 (Lederman *et al.*, 1988). This trial was a prospective study to assess the virologic and immunologic effects of combination anti-retroviral therapy among HIV-1-infected people, where the disease was moderately advanced (Kuritzkes *et al.*, 2000). The HIV-1-infected patients were treated with potent antiviral therapy consisting of ritonavir, lamivudine and azidothymidine. 53 patients were enrolled, but five patients discontinued because of intolerance to the treatment and other problems (Wu and Wu, 2002a). Plasma HIV-1 RNA levels were repeatedly measured with scheduled measurements on days 0, 2, 7, 10, 14, 21 and 28 and at weeks 8, 12, 24 and 48 after initiation of treatment. The lower limit of detection was 100 copies ml^−1^, which was reached within the first weeks of treatment by a considerable proportion of patients. Additional information available for each patient includes measurements of physiological markers taken at baseline and demographic information.

**Fig. 1:**
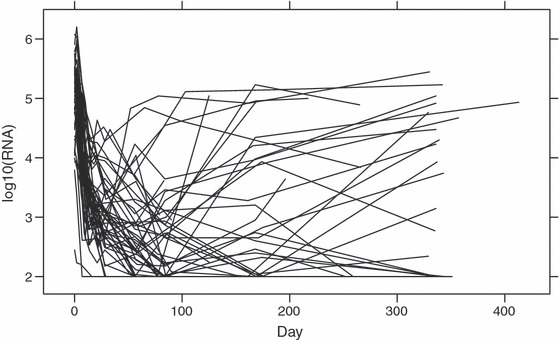
ACTG 315 trial data: logarithmic HIV RNA levels for the 48 patients over time

In total 502 observations from 48 individuals on HIV RNA levels are available from the study, with measurements taken at times ranging from 0 to 413 days since treatment. The amount of censoring was rather high with 16% censored observations. 58% of the patients had at least one censored observation; the average number of censored observations per patient was equal to 1.65. Ignoring the censoring might therefore give biased results and hence the censoring should be accounted for in the fitted model. The development of the HIV RNA levels over time is shown in Fig. 1. The observations from the same individual are joined. Clearly different groups of patients can be distinguished: for some patients the levels decline over time and stay low, whereas for others the levels rise again after a decline at the beginning of the observation period. This implies that a finite mixture model might be necessary to capture these differences. In addition the individual levels also vary and this heterogeneity can be modelled by specifying random intercepts.

The development of the HIV RNA levels over time was modelled by Wu and Ding (1999) using a hierarchical non-linear mixed effects model where they assumed two phases of exponential decay with different rates of decay. Observations censored at 100 were imputed with 50. Extensions of this model which accounted for censoring were presented in Wu (2002), Fitzgerald *et al.* (2002) and Vaida *et al.* (2007). Fitzgerald *et al.* (2002) used multiple imputation, whereas Wu (2002) and Vaida *et al.* (2007) used the expectation–maximization (em) algorithm (Dempster *et al.*, 1977). Different methods to evaluate the e-step were employed in Fitzgerald *et al.* (2002) and Vaida *et al.* (2007). Following Hughes (1999), Wu (2002) used a Monte Carlo method to evaluate the e-step and Vaida *et al.* (2007) proposed a hybrid Monte Carlo and numerical integration em algorithm. As a complementary analysis Wu and Wu (2002b) considered the identification of significant baseline host factors by using demographic and physiological markers to explain interindividual variation in the HIV RNA level development over time.

The application of non-linear mixed effects models assumes that the functional relationship between the explanatory and the dependent variable is known. For dynamic models for HIV RNA levels the assumption is that the logarithmic HIV RNA levels exhibit an exponential decay over time. The rate of decay is in general allowed to vary over time by splitting the observation period into two phases where the decay rate is constant in each phase. This approach allows parsimonious modelling of a complex relationship. However, it imposes a certain structure. In this case it is implied that the HIV RNA levels decrease over time. As an alternative approach this paper considers finite mixtures of mixed effects models with censored data for modelling HIV RNA levels over time. The functional relationship between the independent and dependent variables is assumed to be unknown and is flexibly estimated by using spline regression. The required flexibility of the spline functions as well as the number of groups required to capture the heterogeneity between patients are determined in the model selection step. The mixture model specification allows us to investigate whether the same functional relationship holds for all patients or whether groups of patients with different HIV RNA level developments exist. Hence, this model can either be used

if the *a priori* functional relationship is not known or
to check whether the assumed functional relationship is suitable.

In previous work the need to model interindividual differences was acknowledged by fitting mixed effects models. The model in this paper addresses the problem that interindividual differences might not be sufficiently captured by the random effects. These differences are more flexibly modelled by using a mixture model which allows for distinct groups with different HIV RNA level developments over time. Because the model class proposed also contains the model with only one component, model selection tests whether the models that were used in the previous work sufficiently captured interindividual differences. In addition, one can use the proposed model class to question the assumption of an exponential decay of the levels over time by determining the functional relationship in a data-driven way.

We present the model class of finite mixtures of mixed effects models for censored data together with a suitable estimation method. We outline a fast implementation of the em algorithm. A specific version of the em algorithm for this model class is derived by extending the version proposed for finite mixtures of mixed effects models (see for example Xu and Hedeker (2001)) to allow for censored observations also. We also exploit the fact that formulae for the mean and the variance for a truncated multivariate normal distribution were developed by Tallis (1961) to arrive at a fast implementation as in Vaida and Liu (2009) who developed an em algorithm for fitting linear mixed effects models with censored responses. The resulting em algorithm has the advantage that the expectation (e-)step as well as the maximization (m-)step are given in closed form (if evaluation of the cumulative multivariate normal distribution function is assumed to be given). In addition suitable strategies for model selection and inference are proposed.

An implementation of the estimation method proposed is available in the package flexmix (Leisch, 2004; Grün and Leisch, 2008) for the statistical software environment R (R Development Core Team, 2011). Package flexmix implements a general framework for fitting finite mixtures by using the em algorithm. The main fitting function in the package provides the e-step and all data handling. A suitable m-step for the model class of finite mixtures of mixed effects models with censored data is available through function FLXMRlmmc(). The current release version of flexmix including this functionality is available from the Comprehensive R Archive Network (http://CRAN.R-project.org).

The paper is structured as follows. In Section 2 the model is specified and the estimation and inference are outlined. The details of the em algorithm as well as the derivation of the standard errors are presented in Appendix A. The model is applied to the ACTG 315 HIV RNA level measurements data in Section 3. A simulation study investigating the performance of the proposed methods for estimation and model selection is presented in Section 4. The paper ends with some concluding remarks.

## 2. Finite mixtures of mixed effects models with censored observations

### 2.1. Model specification

Following Laird and Ware (1982) the standard mixed effects model is denoted by


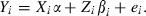



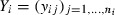
 is the vector of *n*_*i*_ outcomes observed for the *i*th individual. α is the vector of fixed effects and β_*i*_ is the vector of random effects for individual *i*. 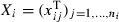 and 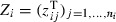 are the corresponding design matrices. *e*_*i*_ is the vector of random errors. β_*i*_ and *e*_*i*_ are assumed to be independent with


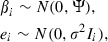


where *N*(μ,Σ) denotes the multivariate normal distribution with mean μ and variance–covariance matrix Σ and *I*_*i*_ is the identity matrix of dimension equal to 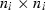. This implies that *Y*_*i*_ follows a multivariate normal distribution with mean 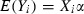 and variance–covariance matrix 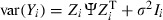 given *X*_*i*_, *Z*_*i*_, α, Ψ and σ^2^.

Extending this model by allowing for a mixture of normals with *G* components implies that


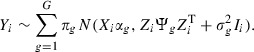


The component weights π_*g*_ are restricted to be positive and sum to 1. The vector of parameters is equal to 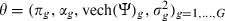. vech(·) is the half-vectorization operator which selects the unique parameters by taking the lower diagonal matrix (including the diagonal). The basic model that was proposed by Laird and Ware (1982) is contained as a special case by setting the number of components *G* to 1.

This model formulation assumes that the component membership is the same over different observations from the same individual, i.e. the mixture distribution accounts for heterogeneity between individuals. A different variant would arise if the component membership also varied within individuals (see for example Yau *et al.* (2003) and Hall and Wang (2005)). However, the resulting model is not a mixture of linear mixed effects models any more, because the random effects are imposed on the mixture and not for each of the components. In addition the evaluation of the e-step is completely different. Another possibility still would be to assume a finite mixture distribution for the random effects, i.e. to estimate the random-effects distribution in a semiparametric way instead of assuming that it follows a normal distribution (see for example Aitkin (1999)).

Whereas mixtures of normal distributions are generically identifiable (Teicher, 1963; Yakowitz and Spragins, 1968), generic identifiability of mixtures of linear regression models depends on the covariate matrix (Hennig, 2000). However, in the case of longitudinal data where the component membership is fixed over repeated observations the condition on the covariate matrix is likely to be fulfilled and, thus, parameter uniqueness for a given mixture distribution to be ensured.

Variants of this general model class could impose equality constraints on some of the parameters over the mixture components. This means that either one, two or all three of

the fixed effects,
the variance–covariance matrices of the random effects and
the variances of the error term

are assumed to be identical over components. For example for the fixed effects this restriction implies that α_*g*_≡α for all *g*. In general only restrictions (b) and (c) will be of interest to check whether a more parsimonious model suitably describes the data when regarding the two variance parameters as nuisance parameters and the fitted component means are in the main focus of the analysis. In what follows only restrictions on the variances are considered.

The model formulation applies only if the values *Y*_*i*_ for each individual are available. However, we assume that we have partly censored data, i.e. instead of *y*_*ij*_ for individual *i* at occasion *j* we observe the pair 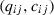, where *q*_*ij*_ is the (possibly censored) response and *c*_*ij*_ is the censoring indicator. In what follows we consider only left-censored observations, i.e. *c*_*ij*_=1 denotes left censoring and *c*_*ij*_=0 indicates that an uncensored response is observed. This can certainly be easily extended to the general case of left, right and interval censoring.

The observed data for each individual *i* are combined to 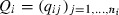 and 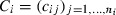. These pairs 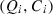 of observed data for each individual are augmented with latent unobserved variables to represent the data-generating process more easily. This leads to the so-called complete data which consist of the uncensored observations *Y*_*i*_ as well as the random effects β_*i*_ and the component assignment indicators γ_*i*_. The component assignment indicators 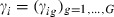 give the component *g* to which an individual belongs and it holds that γ_*ig*_ ∈ {0,1} and 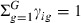 for all *i*.

Hence, we can summarize the observed and the complete data as follows:

observed data, 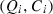 for *i* = 1,…,*N*;
complete data, 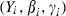 for *i* = 1,…,*N*.

*N* denotes the number of observed individuals.

### 2.2. Estimation

The em algorithm is a general method for maximum likelihood estimation in a missing data setting. The algorithm exploits that in general the complete log-likelihood where missing data are included is easier to maximize than the likelihood where only the observed data are used. It is an iterative method switching between an e-step and an m-step. In the e-step the expected complete log-likelihood given the current parameter estimates and the observed data is determined and the missing data are integrated out. In the m-step the expected complete log-likelihood is maximized to determine new parameter estimates. The new parameter estimates which maximize the expected complete log-likelihood also give a higher value for the likelihood by using only the observed data. If the likelihood is bounded the em algorithm converges, in the sense that the sequence of likelihood values converges to a stationary point (Dempster *et al.*, 1977).

In the case of finite mixtures of mixed effects models with censored data three different kinds of information are missing:

the group assignment γ_*i*_ indicating which component individual *i* is from,
the random effect β_*i*_ and
the observations *y*_*ij*_ of individual *i* and its *j*th observation if *c*_*ij*_=1.

The joint distribution of the complete data *Y*_*i*_, β_*i*_ and γ_*i*_ is given by


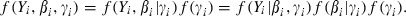


The distribution of *Y*_*i*_ given β_*i*_ and γ_*i*_ with γ_*ig*_=1 is the product of univariate normal distributions for each *y*_*ij*_ with mean 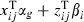 and variance 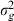. β_*i*_ given γ_*i*_ with γ_*ig*_=1 follows a multivariate normal distribution with zero mean and variance–covariance matrix Ψ_*g*_. Finally, γ_*i*_ follows a multinomial distribution with vector of success probabilities equal to 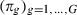. Hence, the complete log-likelihood for individual *i* is up to a constant given by


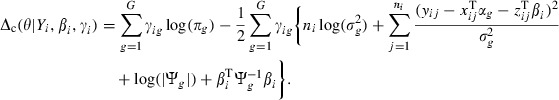
(1)

In the e-step the expectation of the complete log-likelihood that is given in equation (1) is determined conditionally on the current parameter estimates and the observed data. In this step the law of the iterated expectation is used by first only integrating out the random effects and then the underlying values of the censored observations and the component memberships. The mean and the variance of the censored observations can be determined as proposed in Tallis (1961) and Leppard and Tallis (1989) for truncated multivariate normal distributions (see also Vaida and Liu (2009)). In addition to the mean and variance of the truncated multivariate normal distribution the quantities given in equations (3)–(8) in Appendix A need to be determined for the e-step. Given the expected complete log-likelihood the m-step can be solved in closed form. The update for the parameters in the m-step are given by equations (9)–(12) in Appendix A.

The em algorithm proposed naturally extends the proposed algorithm for mixtures of mixed effects models (see for example Xu and Hedeker (2001)). Other versions of the em algorithm which are more in the line of Hughes (1999) or Vaida *et al.* (2007) are also possible. In both Hughes (1999) and Vaida *et al.* (2007), suitable em-type algorithms for mixed effects models with censored data were proposed. The regression coefficients in the m-step are determined by using a linear regression with a general variance–covariance matrix estimated for the random effects, instead of plugging in the expected means of the random effects and using a linear regression where the errors are independently identically distributed. In addition in both Hughes (1999) and Vaida *et al.* (2007) the expected values for the censored observations of each individual were determined by using a Gibbs sampler if the individual has more than two missing observations, instead of using the formulae that were proposed in Tallis (1961) and Leppard and Tallis (1989).

The rate of convergence of the em algorithm depends on the amount of missing data introduced (McLachlan and Krishnan (1977), page 39). Because the variant proposed has three different kinds of missing data it can be assumed that convergence will be slow and that a considerable number of iterations is needed. However, each iteration is quite simple and can be quickly evaluated because all terms are given in closed form. Only efficient evaluation of the cumulative multivariate normal distribution is required. Convergence of the em algorithm is checked by determining the relative change in the log-likelihood of subsequent iterations. The algorithm is stopped if this difference is smaller than an *a priori* specified threshold. The em algorithm is started with an m-step using initial estimates for the group memberships, the random effects and the underlying values of the censored observations. Because convergence is at best to a local maximum, the best result of several runs of the em algorithm with different random initializations is determined to eliminate local maxima and to increase the chance of detecting the global maximum. A comparison of different initialization strategies for finite mixtures of linear and linear mixed effects models was presented in Scharl *et al.* (2010).

### 2.3. Inference

Selecting a suitable model from the model class of finite mixtures of linear mixed effects models with censored data involves deciding on

the number of components,
the covariates in the regressions for the fixed effects,
the covariates in the regressions for the random effects and
constraints on the parameters over the components.

The additional assumption that the covariates are the same for all components considerably reduces the range of possible models.

In general selecting the suitable number of components is a difficult problem in finite mixture modelling which has not yet been completely resolved (see McLachlan and Peel (2000), page 175). A general recommendation for finite mixture models is the Bayesian information criterion (BIC) despite the fact that the regularity conditions do not hold. Nevertheless the BIC has been shown not to underestimate the number of components asymptotically (Leroux, 1992) and to give good results in simulation studies (McLachlan and Peel (2000), page 209).

For finite mixtures of regression models, Wang *et al.* (1996) proposed a two-stage procedure for model selection. In the first stage they selected the optimal number of components *G* among all saturated models according to the BIC. In the second stage the optimal model is chosen among all different component-specific models with the number of components fixed at *G* either by using information criteria or likelihood ratio tests. In addition the second stage can be used to determine whether the random effects and/or residual variances should be restricted to be equal over components. In this second stage model selection methods from the literature on linear mixed effects models can directly be used. To speed up the convergence of the em algorithm for fitting the models that are required in the second stage the algorithm can conveniently be initialized by using the posterior probabilities of the saturated and unrestricted models selected in the first stage.

In the context of finite mixtures of mixed effects models using *B*-splines, Luan and Li (2003) and Ng *et al.* (2006) selected the number of components by using the BIC. In this model selection step they kept the covariates and the random effects fixed. Celeux *et al.* (2005) used different information criteria including Akaike's information criterion (AIC), the BIC and the integrated completed likelihood criterion to select the random effects, constraints over parameters and the number of components. They also did not vary the covariates. Even though comparing different criteria they favour the BIC. A comparison of the performance of the AIC and BIC is presented in a simulation study in Section 4. The results clearly indicate a superior performance of the BIC for selecting the correct number of components. This is in line with previous results and recommendations.

Standard errors for the parameter estimates can be estimated by using the empirical information matrix to perform inference for the selected model. For independent data it is convenient to approximate the empirical information matrix by using only the gradient vector of the log-likelihood functions to avoid calculating second derivatives (see Basford *et al.* (1997)). Equations (13)–(16) in Appendix A indicate how the scores for the component sizes π_*g*_, the fixed effects coefficients α_*g*_, the residual variance 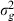 and the variance–covariance matrix of the random effects Ψ_*g*_ are determined.

## 3. Application to the ACTG 315 data

### 3.1. Model estimation and selection

We assume that the functional form of the HIV RNA levels over time is not known and might vary between groups of patients. This model formulation has the advantages that

the application is possible without knowing the functional relationship between dependent and independent variables and
a supposed relationship can be validated.

We use *B*-splines of the time variable with different degrees of freedom as regressors. A similar approach is often pursued for modelling expression levels of microarray experiments over time (see for example Ng *et al.* (2006)). The numbers of degrees in addition to an intercept are varied from 1 to 5 and the degrees of the piecewise polynomial are the minimum of 3 and the specified number of degrees to have at most cubic splines. Different variants are fitted with respect to the residual and the random-effects variances: they are either assumed to be equal over components or different for each of the components. The variation of the degrees of freedom and of the restrictions on the variances lead to 20 different models. For each of these models the number of components is varied from 1 to 5. In addition the same models are also fitted without random effects, i.e. the intercepts are assumed to be identical over respondents.

Each model is fitted with the em algorithm as described in Section 2 by using five different random initializations. The em algorithm is stopped by using the relative change in the log-likelihood as criterion and the threshold is set to 10^−6^. The best model among all these models is selected according to the BIC and the AIC. The BIC selects a mixture model with four components, random intercepts and cubic splines with degrees of freedom equal to 4. For the random-effects equal variances and for the residuals unequal variances are fitted. The AIC prefers a mixture model with five components, random intercepts and cubic splines with degrees of freedom equal to 4. The random-effects variances as well as the residual variances are selected to be not restricted over the components. A comparison of the clusterings that are induced by the posterior probabilities of the two models indicates that for four of the components of the model selected with the AIC a good overlap with the four components of the model selected with the BIC is given. The additional component in the model that is selected with the AIC contains observations which are assigned to three different components in the model that is selected with the BIC.

The two-stage approach for model selection that was proposed in Wang *et al.* (1996) cannot directly be used because it is unclear what the saturated models are if the covariates are *B*-splines of different degrees. However, their approach can be used to select suitable constraints for the random-effects and residual variances. In this case first the best model with respect to the BIC is selected from the models with number of components varied from 1 to 5 and the number of degrees for the *B*-splines varied from 1 to 5 where unrestricted variances for the random effects and the residuals are estimated. In this case in the first step also the model with four components and 4 degrees of freedom for the *B*-splines is selected. This approach would hence lead to the same result because in the second step the restricted model is preferred. Selection with the AIC by using the two-stage approach also leads to the same model because for the AIC no restrictions on the variances are preferred.

### 3.2. Model interpretation

In what follows the mixture model that is selected by the BIC is considered in more detail. This model has four components, random intercepts, cubic splines with degrees of freedom equal to 4, equal variances for the random effects and unequal variances for the residuals. This follows the general recommendation to use the BIC for model selection. The component sizes that were obtained are equal to 0.32, 0.26, 0.21 and 0.21. The numbers of patients who were assigned to each component according to the maximum *a posteriori* probabilities are 16, 12, 10 and 10 respectively. The assignment of patients to the various components is unambiguous for most patients. The mean maximum *a posteriori* probability is 0.96 and 90% of the patients have a maximum *a posteriori* probability of at least 0.86.

**Fig. 2:**
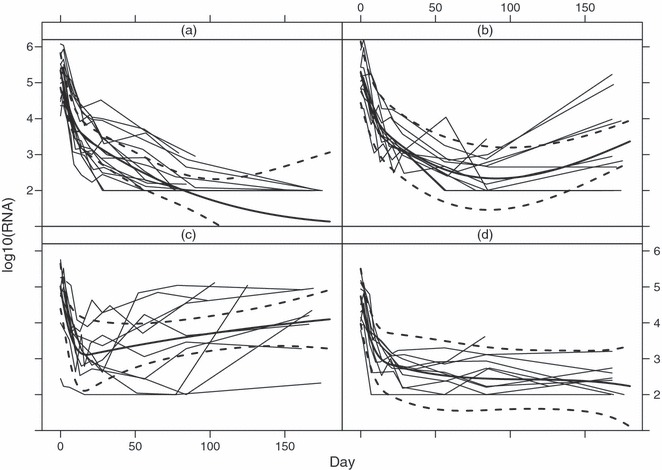
Logarithmic HIV RNA levels for the 48 patients over time separately for each component and the fitted component mean (with the 95% confidence interval): (a) component 1; (b) component 2; (c) component 3; (d) component 4

The HIV RNA levels of the patients split into four groups according to the component assignments are given in Fig. 2. In each panel the estimated means of the corresponding component (with 95% confidence intervals) are also shown. Fig. 2 indicates that for patients from components 1 and 4 the expected behaviour, the decrease in RNA level over time which levels off, is observed. These two components could also be modelled by using the non-linear mixed effects framework where the functional form is supposed to be a linear combination of two exponential functions as in Vaida *et al.* (2007). However, components 2 and 3 have a different behaviour. After a decrease at the beginning of the observation period, the levels of RNA increase again later. Fitting this kind of functional behaviour would not be possible if it is fixed *a priori* to be a linear combination of two decreasing functions. Fig. 2 also indicates that the uncertainty in estimating the means is higher for censored observations than for uncensored.

For each of the components a strong decay in HIV RNA levels is observed at the beginning of the observation period. The length of this period of strong decay differs over components as well as how HIV RNA levels change in the second stage. The end of this first time period could be identified as the time of viral rebound for the second and third component where the HIV RNA levels increase in the following period. Viral rebound occurs very soon after the beginning of treatment for patients in component 3. For component 1 the levels decrease over the whole observation period; the amount of decrease grows smaller only over time. For component 4 after the first stage of decay the HIV RNA levels are stable over the remaining observation period. This clearly shows that accounting for these differences in HIV RNA developments over time allows for improved estimation of viral rebound which was for example also investigated by using non-linear mixed effects models with censoring in Fitzgerald *et al.* (2002).

Predicting *a priori* from baseline host factors the component membership of patients is of interest

to explain interindividual differences based on these variables and
to forecast the viral load trajectories of the patient for improved clinical decisions and individualized treatments.

**Fig. 3:**
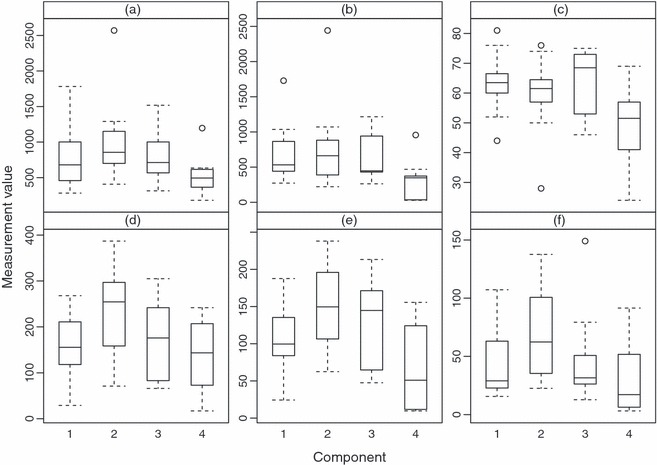
Parallel boxplots of the baseline host factors which differ significantly at the 5% level of significance for the components induced by the mixture model: (a) CD8 cells; (b) CD838 cells; (c) pCD8 cells; (d) CD4 cells; (e) CD438 cells; (f) CD438dr cells

31 demographic variables and physiological markers have been measured at baseline in the clinical trial ACTG 315. The data were described in Wu and Wu (2002b) where the variables are listed and explained in Table 1. A Kruskal–Wallis rank sum test is used to check for significant differences in the baseline covariates between the components. This procedure identifies six baseline covariates which differ significantly at the 5% level of significance between the components. Parallel boxplots for the components for each of the variables are given in Fig. 3. All covariates identified are related to CD8 cells. This is different from Wu and Wu (2002b) who in addition to CD8-cell-related covariates also selected the baseline RNA level and covariates that are related to CD4 cells. However, Wu and Wu (2002b) used the baseline host factors to identify variables where values of the covariates are associated with the (individual) decay rates. By contrast, our approach identifies covariates where patients have similar values who also have a similar HIV RNA level development pattern over time.

**Table 1:** Selected models for the 100 bootstrap samples

Criterion	Degrees of freedom	Variance		Results for the following numbers of components			
			
		Residuals	Random effects	2	3	4	5
AIC	4	Unequal	Unequal	0	0	3	14
	4	Unequal	Equal	0	0	29	15
	5	Unequal	Unequal	0	0	5	6
	5	Unequal	Equal	0	0	21	7
BIC	4	Unequal	Unequal	0	0	1	0
	4	Unequal	Equal	0	13	53	0
	5	Unequal	Unequal	1	0	0	0
	5	Unequal	Equal	0	11	20	0
	5	Equal	Equal	0	0	1	0

To investigate further the differences detected, pairwise comparisons using a Wilcoxon rank sum test are performed, where the *p*-values are adjusted for multiple testing by using Holm's method and a level of significance of 5% is used. For CD8 cells significant differences are detected between components 2 and 4. For CD838 cells significant differences are detected between components 1 and 4 and components 2 and 4. For pCD8 cells significant differences are detected between components 1 and 4. This indicates that component 4 significantly differs from the other components on the basis of these baseline host factors. For component 3, no covariate can be identified which exhibits a significant difference from any other component.

## 4. Simulation study

The performance of the model estimation and selection strategy proposed is evaluated by using the parametric bootstrap. This allows us to check whether the available data are sufficient to estimate the different groups in the data reliably. 100 samples are drawn from the fitted model to the HIV RNA data in Section 3 which was selected as the best model by using the BIC. The models are estimated in the same way as in Section 3, i.e. the number of components and the number of degrees of freedom are varied from 1 to 5 and the variances of the residuals as well as of the random effects are either restricted to be the same over components or are allowed to differ.

Model selection is performed by choosing the best model according to the AIC and the BIC from all models that were fitted to each bootstrap sample. The models selected are given in Table 1. The BIC clearly performs better than the AIC by choosing the correct model for 53% of the bootstrap samples whereas this is only so for 29% for the AIC. For the BIC the number of components is only underestimated, but never overestimated. The reverse is true for the AIC. The numbers of individuals are quite small for each component. Random sampling from the mixture distribution therefore can easily lead to samples where only very few individuals are drawn from one component and which is therefore not identified as a component when using the BIC. The correct number of degrees of freedom is also selected for 67% of the bootstrap samples when using the BIC. For the AIC for 61% of the samples the correct number of degrees of freedom is selected and otherwise the flexibility of the spline functions is overestimated. The AIC and BIC values differ only slightly between the true model and the selected model. The BIC values are only at most 3.3% smaller for the selected models for 80% of the cases and the AIC values of the selected models are only at most 5.6% smaller for 80% of the cases.

**Fig. 4:**
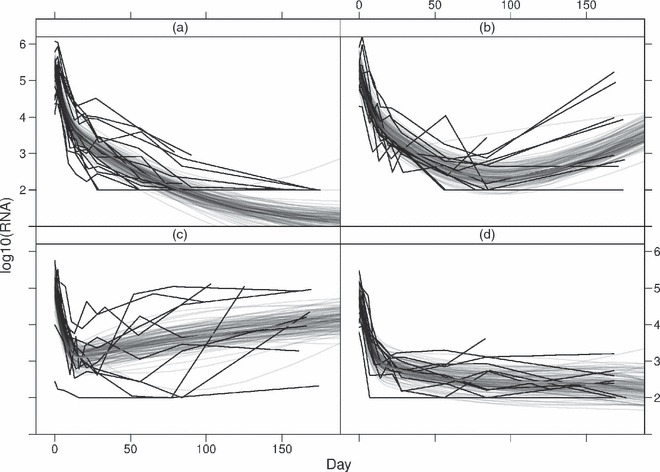
Logarithmic HIV RNA levels for the 48 patients over time separately for each component and the fitted component means for the bootstrap samples when selecting the correct model: (a) component 1; (b) component 2; (c) component 3; (d) component 4

The estimated means for each component are compared if the correct model is selected. The components are matched to maximize the agreement in the component assignment. Fig. 4 shows the data split according to the model selected in Section 3. The estimated means of the components of the original model are given together with the component means of the models fitted to the bootstrap samples. A strong correspondence is observable to the 95% confidence intervals estimated by relying on standard asymptotic theory as indicated in Fig. 2. This indicates that the results by using standard asymptotic theory are reliable and the additional computational effort of using the parametric bootstrap does not pay off.

The concordance between the component assignments determined for the ACTG 315 data by using the original model and the correct model fitted to the bootstrap samples is evaluated by using the Rand index corrected for chance (Hubert and Arabie, 1985). The mean corrected Rand index over the 100 samples is 0.91 with a standard deviation of 0.09. The minimum Rand index corrected for chance is 0.51 and the maximum Rand index corrected for chance of 1 is attained 25 times.

The simulation study indicates that the model that was fitted to the data is reasonable and can be re-estimated surprisingly well by using data sets of the same size drawn from the fitted model. In addition it indicates the superior performance of the BIC if the true number of latent groups in the data is of interest.

## 5. Concluding remarks

An alternative approach for dynamic modelling of HIV RNA levels is proposed by using finite mixtures of linear mixed effects models with censored data. A suitable em algorithm for fitting this model class in a frequentist setting is derived and possible methods for inference are discussed. In contrast with previous approaches for fitting mixed effects models with censored data by using the em algorithm, no Gibbs sampling is involved for determining the means and variances of the censored observations (Hughes, 1999; Vaida *et al.*, 2007). The closed form solutions that were given in Tallis (1961) and Leppard and Tallis (1989) are used as in Vaida and Liu (2009). This requires only evaluating the cumulative multivariate normal distribution function which can be efficiently done by using the method that was proposed in Genz (1992).

The application indicates that assuming that the functional relationship is known between the dependent and the independent variables might lead to overly restrictive model specifications. The model class proposed constitutes an addition to the available modelling toolbox which allows us to check assumptions and to investigate alternative models. Because the model is a direct extension and generalization of previously proposed approaches, model selection can easily be integrated and a suitable model can be chosen.

Further possible extensions of the presented model class include also to relax the assumption of normality for the remaining errors. For example, finite mixtures of multivariate *t*-distributions were previously used for robust mixture modelling and the error distribution could therefore be replaced by a *t*-distribution. Following Peel and McLachlan (2000) the *t*-distribution could be interpreted as a normal scale mixture model and in the future we would like to attempt to adapt the EM algorithm for this model by including a latent gamma-distributed variable.

Using spline functions to model an unkown functional relationship is appealing because of its flexibility and the possibility of choosing the necessary amount of smoothing by using model selection methods. For finite mixtures, different numbers of degrees of freedom could be used for each of the components. This would allow us to model components suitably where the degree of smoothness varies considerably. For the present application this did not seem to be an issue and the same amount of flexibility for the spline functions was suitable for each component. Relaxing the assumption of equality of smoothness in the components would in general substantially increase the model space and fitting all different models would be infeasible. This could be solved by employing two different approaches:

a two-step approach where the other aspects of model selection are determined in the first step and kept fixed in the second step where the degree of flexbility in each of the components is determined and
by including the determination of suitable degrees of freedom in the em algorithm.

Both possible approaches should be investigated in future work.

## Appendix A: Data and computational details

The data were downloaded as ACTG315LongTermViralDynamicsData.cfm and ACTG315LongitudinalDataNLMEData2.cfm from the data set collection at http://www.urmc.rochester. edu/smd/biostat/people/faculty/WuSite/.

All computations are done in R by using package flexmix and the new model driver FLXMRlmmc(). For determining the mean and the covariance of the censored observations the algorithm that was proposed in Leppard and Tallis (1989) was implemented by using package mvtnorm (Genz, 1992). Numerical problems arise for very small probabilities, e.g. for observations from different components. To avoid these problems observations with a small posterior probability (smaller than or equal to 10^−6^) are omitted in the m-step of this component. In addition, if infeasible results are obtained by using mvtnorm, they are replaced by a rough, but feasible, alternative estimate.

### Appendix B: Details on estimation and inference

#### B.1. EM algorithm

For convenience of notation the subscripts u (uncensored) and c (censored) are used for any vector of observations from individual *i* to define the subvectors where *c*_*ij*_=0 and *c*_*ij*_=1 respectively. This signifies for example that

In addition **1**_c,*i*_ indicates a vector of 1s with length equal to  and *I*_c,*i*_ the identity matrix of the same number of rows and columns. This notation also implies that

*Y*_c,*i*_ given *Y*_u,*i*_ (or equivalently *Q*_u,*i*_) and γ_*ig*_=1 follows a multivariate Gaussian distribution with mean and variance

This implies that the distribution of *Y*_c,*i*_ given  and γ_*ig*_=1 is a truncated multivariate Gaussian distribution with upper bound *Q*_c,*i*_. Formulae for deriving the mean and the variance for a multivariate Gaussian distribution truncated from below were given in Tallis (1961) and Leppard and Tallis (1989) and can easily be analogously derived for a multivariate Gaussian distribution truncated from above. We denote these means and covariances by

The analogously defined  is equal to *Y*_u,*i*_ and  is equal to a matrix of 0s. In the e-step the expectation of the complete log-likelihood with respect to the current parameter estimates θ given the observed data is determined. This integrates out the missing data. The expectation is iteratively determined by first eliminating the random effects and determining

and

using  (see also Xu and Hedeker (2001)).

The expectation with respect to θ conditional on  is then

(2)

The various terms that are needed in equation (2) are

(3)

(4)

(5)

(6)

(7)

(8)

The analogously defined ψ_u,*ig*_ is equal to a matrix of 0s. Φ(*x*;μ,Σ) and φ(*x*;μ,Σ) denote the cumulative distribution function and the density function of the multivariate normal distribution with mean μ and variance–covariance matrix Σ evaluated at *x*. diag(*x*) gives a diagonal matrix with *x* in the diagonal.

The m-step consists of determining the first derivatives of the expected complete log-likelihood given the data and the current parameter estimates with respect to the parameters and setting them equal to 0. The solution of these score functions is given in closed form.

(9)

(10)

(11)

(12)

These formulae can be easily modified to estimate fixed variances for the residuals or fixed variance–covariance matrices for the random effects over components by weighted averaging over the component-specific parameter estimates.

#### B. 2. Provision of standard errors

In the case where observations are independent and identically distributed the information matrix can be approximated by the empirical information matrix (Basford *et al.*, 1997; McLachlan and Peel, 2000). The empirical information matrix is equal to

where

The scores for π_*g*_ are determined for *g* = 1,…,*G*−1.

(13)

The scores for the other parameters are determined for *g* = 1,…,*G*.

(14)

(15)

(16)

Again these formulae can be easily modified when fixed variances for the residuals or fixed variance–covariance matrices for the random effects over components are estimated by weighted averaging over the scores of the component-specific parameters.
